# Rapid symptomatic relief of HER2-positive gastric cancer leptomeningeal carcinomatosis with lapatinib, trastuzumab and capecitabine: a case report

**DOI:** 10.1186/s12885-018-4116-0

**Published:** 2018-02-20

**Authors:** Xiao-Dong Jiao, Chunming Ding, Yuan-Sheng Zang, Guanzhen Yu

**Affiliations:** 1 Department of Medical Oncology, Changzheng Hospital, Shanghai, China; 2 School of Laboratory Medicine and Life Science, Wenzhou Medical University, Zhejiang, China; 3 Department of Oncology, Longhua Hospital Affiliated to Shanghai University of Traditional Chinese Medicine, Shanghai, China

**Keywords:** Gastric cancer, Leptomeningeal Carcinomatosis, HER2, Lapatinib

## Abstract

**Background:**

Gastric cancer patients with widespread metastasis, especially meningeal metastases, have an extremely prognosis and limited therapeutic choices.

**Case presentation:**

We reported the case of a 39-year-old male patient with HER2-positive gastric cancer with bone and meningeal metastases. He presented with multiple bone metastases and received 3 cycles of docetaxel plus S1. However, he complained with headache and imaging examinations revealed leptomeningeal carcinomatosis. FISH revealed that tumor cells in the cerebrospinal fluid were HER-positive. Herceptin was added to the regimen, but the symptoms were not relieved, the patient suffered from dizziness and nausea. The chemotherapy regimen was switched d to lapatinib (orally at 1250 mg/day, every day), capecitabine (orally at 1000 mg/m2, bid for 2 weeks, followed by a 1-week rest interval, as 1 cycle) and Herceptin (390 mg/3 weeks). After 3 weeks of the new treatment, all the symptoms relieved. The clinical complete response was maintained for 3 months.

**Conclusions:**

Lapatinib/Capecitabine combination therapy is an alternative treatment strategy for leptomeningeal carcinomatosis of HER2-positive gastric cancer in which trastuzumab and/or chemotherapy essentially has no effect.

## Background

Gastric cancer is the third leading cause of cancer-related death in the world [1]. It is estimated that more than 60% gastric cancer patients harbored lymph node metastasis and/or distant metastasis [2]. Although advances in the use of combination chemotherapy regimens, the prognosis for metastatic gastric cancer (MGC) is often poor, the median overall survival (OS) less than one year [3]. Recently, the ToGA study demonstrated that targeting human epithelial growth factor receptor 2 (HER2), combined with chemotherapy, prolonged OS to 13.8 months in advanced gastric or gastro-esophageal junction cancer [4]. Trastuzumab plus chemotherapy is currently regarded as the first-line standard of care for HER2-positive MGC. However, it has several shortcomings: the development of resistance and limited ability to cross the blood-brain barrier due to its large molecular weight [5]. Therefore, a small molecule inhibitor of HER2 and the epidermal growth factor receptor [EGFR] (lapatinib) has been noted to be a promising agent for HER2-positive patients suffering from brain metastasis [6].

Here we report a case of metastatic HER2-positive gastric cancer. Most importantly, the patient developed vermis leptomeningeal carcinomatosis, a rare complication of gastric cancer with extremely poor outcome. The therapy was switched to capecitabine with dual HER2 blockade (trastuzumab and lapatinib), and intrathecal injection of methotrexate and dexamethasone. The patient responded remarkably well to this regimen, with relieved symptoms including headache, nausea, vomiting, neck resistance, gait disturbance, etc..

## Case presentation

**Fig. 1:**
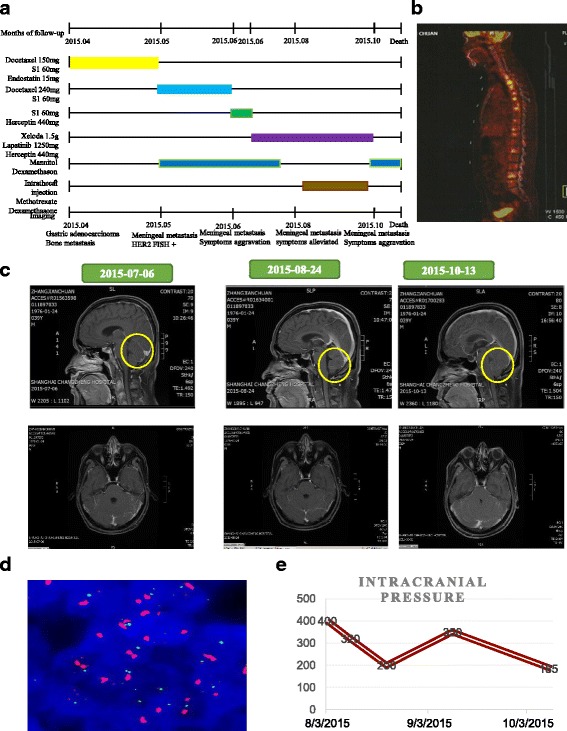
The course of treatment. **a** Timeline describing clinical course, treatments administered and selected imaging assessments. **b** Positron emission tomography-computed tomography (PET/CT) scan demonstrated widely metastatic disease throughout his skeleton. **c** MRI of the brain showed edema of cerebellum at indicated time point. **d** HER2 testing of these tumor cells by fluorescent in-situ hybridization (FISH). **e**, The changes of intracranial pressure during the treatment process

The clinical course was presented in Fig. 1a. A 39-year-old Chinese man presented with swelling abdomen and a high level of CEA: 465 ng/ml. Electronic gastroscopy and biopsy confirmed poorly-differentiated adenocarcinoma, mixed with ring cell carcinoma, in the distal stomach. Further workup with positron emission tomography-computed tomography (PET/CT) scan demonstrated widely metastatic disease throughout his skeleton (Fig. 1b). On 2015–4-13, he was initially treated with docetaxel (150 mg, d1, q2w), S1 (orally 60 mg, Bid, d1–10, q2w), and Endostatin (15 mg, d1–7, q2w) for two cycles, followed by docetaxel (240 mg, d1, q3w), S1 (orally 60 mg, Bid, d1–10, q3w) for one cycle (2015–5-20). During the third cycle of therapy, he complained positional headache, nausea, and vomiting. He stated that the headache was located in the back side of head and was associated with standing up from a lying or sitting position. The above symptoms would relieve once he returned to a supine position. He denied vision changes, gait difficulties from weakness or ataxia, memory problems, sensory abnormalities. Physical examination revealed stable vital signs with nuchal rigidity. There was no evidence of other neurologic deficits. The patient underwent MRI of the brain and the scan revealed vermis cerebelli meningeal metastasis and edema in the vermis cerebelli (Fig. 1c). The initial lumbar puncture demonstrated a high intracranial pressure (data unknown) and found adenocarcinoma cells. HER2 testing of these tumor cells was positive by fluorescent in-situ hybridization (FISH) (Fig. 1d). Therefore, the patient was diagnosed as metastatic leptomeningeal carcinomatosis. Leptomeningeal carcinomatosis is often found in advanced gastric cancer of signet cell pathology, as in this case. On 2015–6-19, his treatment was switched to S1 and Herceptin. 2 days later, he developed vision changes and lethargy. After discussion at multi-disciplinary tumor board, on 2015–7-1, his treatment was switched to capecitabine, trastuzumab, and lapatinib. A month later, the general condition of this patient improved dramatically, and CEA in the plasma decreased notably (Table 1). Intrathecal injection of methotrexate and dexamethasone was administrated for 4 times (2015–8-3, 8–10, 8–21, and 9–10). The pressure of CSF continuously decreased when intrathecal injection was given, CEA in CSF was stable during this period (Table 2). He tolerated well and headache gradually relieved.

**Table 1:** CEA levels in the blood

	2015–06-30	2015–07-30	2015–08-07	2015–08-20	2015–09-08	2015–10-09
CEA(μg/L)	804.7	115.3	71.3	39.13	24	26.69

**Table 2:** Components in cerebrospinal fluid of the patient with leptomeningeal carcinomatosis along with treatment of this disease

DATE	2015–08-03	2015–08-10	2015–08-21	2015–09-10	2015–10-10
Intracranial pressure(mmH_2_O)	>400	320	200	350	185
Glucose(mmol/L)	3.3	2.9	3.3	3.4	3.8
Albumen(mg/L)	525	534	552	585	494
Chloride(mmol/L)	100	110	132	115	98
CEA(μg/L)	78.12	78.94	75.03	NA	26.69

Along with the treatment, the whole condition of this patient turned well, intracranial pressure decreased (Fig. 1e and Table 2), nuchal rigidity dismissed, and headache relieved. Unfortunately, on October 1, he suddenly developed severe gait difficulties from ataxia, followed by unrelieved headache with vomiting, diplopia, and difficulty with speech. MRI brain revealed extremely swelling vermis cerebelli (Fig. 1c). He was taken to the emergency room and the neurosurgeon suggested surgical intervention. Given the poor outcome of this disease, the patient’s family refused surgical intervention and suggested drugs to palliate his symptoms. However, the patient’s performance status deteriorated progressively and died soon.

**Fig. 2:**
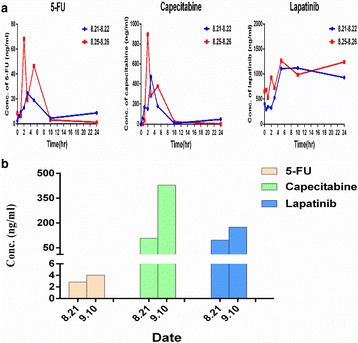
The concentrations of 5-Fu, capecitabine, and lapatinib in peripheral blood and cerebro-spinal fluid (CSF). **a** The distribution of 5-Fu, capecitabine, and lapatinib in peripheral blood after administrated with capecitabine and lapatinib. **b** The concentrations of capecitabine and lapatinib in the CSF

During the process of the treatment, we monitored the concentrations of 5-Fu, capecitabine, and lapatinib in the blood and cerebro-spinal fluid (CSF). Figure 2a showed that the levels of 5-Fu and capecitabine reached a peak 2 h after administration and declined to normal level at 10 h, while lapatinib maintained a high level in peripheral blood. Importantly, the concentrations of capecitabine and lapatinib both maintained a relatively high level in the CSF when these drugs were administrated (Fig. 2b).

## Discussion and conclusions

Leptomeningeal carcinomatosis (LC) is a rare complication of solid tumors in which the disease spreads to the membranes (meninges) of the central nervous system [7]. LC occurs in approximately 5% of people with cancer, more common in breast, lung cancers and melanoma [8]. Very few patients with gastric cancer can develop leptomeningeal metastasis, with estimated rates of 0.16% to 0.69% [9, 10]. LC is more often observed in advanced gastric cancer of signet cell pathology, just as in this case. Due to increased intracranial pressure, most patients with LC present with severe neurologic manifestations, including headaches (usually associated with nausea, vomiting, light-headedness), gait difficulties from weakness or ataxia, memory problems, and sensory abnormalities, most of which could be observed in our patient [9]. In addition to elevated opening pressure on lumbar puncture, the protein level was elevated and the glucose level was lowed in the CSF, as in this patients [11]. MRI is a superior choice in diagnosing this disease, while CSF cytology will be necessary to confirm LC pathologically. As for our patient, MRI and CSF cytology both proved leptomeningeal carcinomatosis.

The prognosis of LC is extremely poor, if left untreated, median survival is 4–6 weeks; if treated, median survival is 2–3 months [12]. Meanwhile, the treatment of LC is limited and intrathecal chemotherapy (methotrexate) is often suggested, but without definite curative effect, as in this patient. Interestingly, our patient presented with HER2-positive LC. The combination of trastuzumab with chemotherapy prolonged the median OS of advanced gastroesophageal cancer from under 12 to 13.8 months [4]. Because of de novo resistance and acquired resistance, only part of the patients benefited from trastuzumab therapy and the median progression-free survival (PFS) was merely 6.7 months [4, 13]. There are no evidences to guide clinical practice regarding trastuzumab-based chemotherapy in gastric cancer with LC. HER2-positvie breast cancer with brain metastasis appears to derive benefit from lapatinib plus capecitabine therapy [14]. Median overall survival (OS) in patients treated with lapatinib plus capecitabine was higher than in patients treated with trastuzumab-based therapy (19.1 vs. 12 months). Previous trials confirmed the safety and efficiency of the combination of trastuzumab and lapatinib in treatment-refractory patients with HER2-positive metastatic cancer [15]. A synergistic effect between lapatinib and trastuzumab against HER2-positive gastroesophageal cancer has been observed in vivo and in vitro [16]. Based on these evidences, our patient was administrated with capecitabine combined with both trastuzumab and lapatinib. He tolerated and responded well to this treatment. All the symptoms induced by LC alleviated and sustained for more than three months. To the best of our knowledge, this is the first report with dual-targeted therapy with trastuzumab and lapatinib in treating gastric cancer with LC.

There are rare experiences in the treatment of meningeal carcinomatosis. The high levels of lapatinib concentration in peripheral blood and CSF gave the direct evidence that our patient responded well to lapatinib-based chemotherapy. Unfortunately, the concentration of lapatinib in CSF is only about 1/5 of that in blood. This data is consistent with previous findings that average lapatinib concentration in brain metastases was only 10–20% of those in peripheral metastases [17]. Intrathecal administration of trastuzumab seems to be a safe and in some cases effective option for those HER2-positive metastatic breast cancer [18]. Our patient deteriorated rapidly when he acquired resistance to lapatinib and we didn’t have to chance to carry out this option. Considering the fact that CEA level and intracranial pressure were much less than before, it is doubtable whether this patient died of acquiring resistance to HER2-targeted therapy. If so, the potential mechanisms underlying resistant to trastuzumab have been well-discussed: alteration in the HER2 receptor, loss of the tumor suppressor phosphatase and tensin homolog (PTEN), activation of various signaling pathways, immune response, etc. [19]. Whether lapatinib-resistance has the same mechanisms as trastuzumab-resistance need further study [20].

In summary, we report a case of HER2-positive gastric adenocarcinoma with rare leptomeningeal carcinomatosis and intrinsic resistance to trastuzumab-based therapy. Dual HER2 blockade appeared to be very effective in preventing LC progression. Unfortunately, the disease progressed eventually even the concentration of lapatinib sustained a relative high level. More strategies should be explored to target LC and to increase the concentration of specific drugs.

## Abbreviations

CSF: Cerebro-spinal fluid
HER2: Human epithelial growth factor receptor 2
LC: Leptomeningeal carcinomatosis
MGC: Gastric cancer
OS: Overall survival
PET/CT: Positron emission tomography-computed tomography
